# P89 Decreasing delays in infant urine collection with a bladder stimulation technique (Dangle-Tap)

**DOI:** 10.1093/jacamr/dlaf230.096

**Published:** 2025-12-04

**Authors:** Claire Crouch, Ruth Cantwell, Victoria Austin, Andrew McArdle

**Affiliations:** Alder Hey Children’s NHS Foundation Trust, Liverpool, UK; Alder Hey Children’s NHS Foundation Trust, Liverpool, UK; Alder Hey Children’s NHS Foundation Trust, Liverpool, UK; Alder Hey Children’s NHS Foundation Trust, Liverpool, UK

## Abstract

**Background and methods:**

BSAC suggests 10%–20% of febrile infants presenting to the ED will have UTI. Prompt identification of the source of infection enables timely antibiotic cessation or targeting of therapy to appropriately treat the identified infection whilst also reducing the risk of driving antimicrobial resistance. Delay in collecting high quality urine samples can lead to undertreatment or prolonged empirical therapy. During Antimicrobial Stewardship (AMS) rounds at Alder Hey Children’s Hospital, we noted that urine collection in infants under 90 days admitted with sepsis concerns was frequently delayed beyond initial antibiotic administration. Delays in spontaneous voiding and avoidance of invasive techniques were felt to contribute. We investigated the impact of these delays and implemented an educational intervention to prioritize efficient collection using a published bladder stimulation technique (‘Dangle Tap’).

**Results:**

The technique was successfully introduced to a busy paediatric acute medical ward. 44 nurses were trained. 26 (59%) of trained staff completed a post technique survey. 84% (16/19) reported having successfully used it to collect a urine sample (7/26 had had no opportunity to use the technique at the time of surveying). When successful, staff reported voiding occurred in less than 5 min in all but one case (which was reported as 5–8 min). All respondents would recommend using the technique. Where failures were experienced, factors perceived to contribute were dehydration, poor feeding and the procedure not being well tolerated. We reviewed 453 urine samples sent from febrile infants <90 days between March 2024 and February 2025. 51 samples had microscopic evidence of UTI (>100 white cells per microlitre). 15/26 (58%) samples taken before antibiotics had pure growth of an organism, compared to 2/20 (10%) from samples post-antibiotics.

**Conclusions:**

Delays in urine collection in febrile infants under 90 days are common, and collection after antibiotic administration dramatically impedes growth, with significant clinical implications. Early experience in Alder Hey supports positive experience of staff and the reported success rate is high. Work in progress will evaluate impact on timing of urine collection, potential false negative culture rates and impacts on antimicrobial stewardship.

